# Impact of Axillary Burden on Survival: A Comparative Study of Invasive Lobular Carcinoma and Invasive Ductal Carcinoma in Early-Stage Breast Cancer

**DOI:** 10.3390/cancers17061002

**Published:** 2025-03-17

**Authors:** Kwang Hyun Yoon, Jee Hyun Ahn, Jee Ye Kim, Hyung Seok Park, Seung Il Kim, Seho Park

**Affiliations:** 1 Department of Surgery, Gangneung Asan Medical Center, Gangneung 25440, Republic of Korea; 0415735@gmail.com; 2 Yonsei University Graduate School of Medicine, Seoul 03722, Republic of Korea; 3 Division of Breast Surgery, Department of Surgery, Severance Hospital, Yonsei University College of Medicine, Seoul 03722, Republic of Korea; jhahn35@yuhs.ac (J.H.A.); jeeye0531@yuhs.ac (J.Y.K.); imgenius@yuhs.ac (H.S.P.); skim@yuhs.ac (S.I.K.)

**Keywords:** breast neoplasms, lymphatic metastasis, invasive lobular carcinoma, invasive ductal carcinoma, survival

## Abstract

Purpose: Invasive ductal carcinoma (IDC) and invasive lobular carcinoma (ILC) are the most common breast cancer types. While they differ biologically and pathologically, their association with axillary lymph node (ALN) metastasis and survival remains unclear. This study compares the clinical features of ILC and IDC to evaluate ALN surgery considerations for ILC patients. Materials and Methods: We retrospectively analyzed 3543 patients who underwent upfront surgery for early breast cancer at Yonsei University Severance Hospital between January 2015 and December 2019. Multivariate logistic regression assessed factors linked to ALN metastasis, while Cox regression identified predictors of recurrence and survival. Results: Among the patients, 92.1% had IDC and 7.9% had ILC. T2-stage tumors were more prevalent in ILC (31.4% vs. 18.1%, *p* < 0.001). The rates of ALN metastasis were similar between the groups (IDC: 21.1%, ILC: 24.6%, *p* = 0.655); however, the presence of more than two metastatic ALNs was more frequent in ILC (9.6% vs. 5.0%, *p* = 0.004). Factors associated with having >2 metastatic ALNs included histology, suspicious axillary ultrasound, T stage, and lymphovascular invasion. The median follow-up period was 65 months, with no significant differences observed in 8-year recurrence-free survival (ILC: 95.2%, IDC: 94.1%, *p* = 0.134) or 5-year overall survival (ILC: 97.1%, IDC: 97.4%, *p* = 0.289). Conclusions: ILC features larger tumors and a higher nodal burden but has similar survival rates to IDC with proper treatment. Caution is essential in axillary surgery to avoid underestimating the nodal burden.

## Simple Summary

Breast cancer is classified into invasive ductal carcinoma (IDC) and invasive lobular carcinoma (ILC), with these two types being the most common. In a study on early breast cancer, ILC showed more advanced stages, but lower levels of poor prognosis biomarkers compared to IDC. Despite differences in pathological staging, appropriate treatment resulted in no significant difference in survival rates between the two types. These findings suggest the potential to improve quality of life without compromising survival. This study highlights the importance of personalized treatment strategies in breast cancer to reduce overtreatment and enhance patient well-being.

## 1. Introduction

Breast cancer treatment is customized for each individual, considering factors such as the stage, subtype, age, and other relevant factors. Surgery, the most crucial and foundational treatment for breast cancer, is also personalized. During Halsted’s time, surgical outcomes varied based on the extent and nuances of the procedure, leading to the widespread adoption of radical mastectomy [1]. Following Fisher’s groundbreaking revelations, breast cancer began to be understood not just as a localized disease, but as a systemic one, underscoring the importance of integrating chemotherapy and radiotherapy with surgical approaches [2]. This insight shifted the focus from radical mastectomy to breast-conserving surgery. Sentinel lymph node biopsy (SLNB) was introduced to axillary surgery in breast cancer, reducing the complications associated with axillary lymph node dissection (ALND). Building on the evidence from the Z0011 trial, ALND is sometimes omitted even in cases of metastasis, depending on the preoperative axillary status [3]. Thus, surgical approaches are further tailored based on preoperative axillary imaging findings or clinical staging, with a growing emphasis on less invasive techniques that achieve comparable survival rates while reducing harm.

Invasive ductal carcinoma (IDC) and invasive lobular carcinoma (ILC) account for approximately 80–85% and 10–15% of all breast cancers, respectively [4]. Pathologically, IDC originates from the ducts and forms a mass, making it relatively easier to detect through imaging studies. In contrast, ILC starts in the lobules and spreads in a single-file pattern, making the tumor borders indistinct and not forming a clear mass, complicating detection through imaging [5,6,7]. Consequently, ILC is often diagnosed at larger sizes and is more likely to be multifocal and bilateral [8,9,10]. ILC differs significantly from IDC in transcriptomic profiles, particularly in metastatic patterns, even when matched for grade and molecular subtype. These findings highlight that ILC and IDC are distinct diseases, requiring tailored diagnostic and therapeutic approaches [11,12]. Thus, there are distinct differences in the patterns of axillary lymph node (ALN) metastasis. The axillary burden is a critical consideration in managing breast cancer, as it significantly impacts prognosis, treatment choices, and oncological outcomes. These histological differences substantially affect the biological behavior of tumors and their propensity for metastasis, highlighting the importance of considering whether tailored surgery is advisable based on these distinctions.

This study analyzes the influence of biological and pathological differences between ILC and IDC based on patterns of ALN metastasis and associated survival outcomes. Building on this, the study aims to determine whether de-escalation of axillary surgery is feasible even in cases of ILC.

## 2. Materials and Methods

### 2.1. Patient Selection and Clinicopathological Characteristics

Patients with primary IDC and ILC who underwent upfront surgery between January 2015 and December 2019 were retrospectively selected from the medical databases of Yonsei University Severance Hospital in Seoul, Korea. The exclusion criteria included de novo stage IV disease, pathological Tis, preoperative clinical T3 or higher, neoadjuvant chemotherapy, cancers of non-epithelial origin, occult breast cancer, and absence of axillary surgery. The medical database documented key characteristics such as age at diagnosis and postoperative pathological findings. These findings included histological type; nuclear grade; histological grade (HG); tumor size; extent of lymph node involvement; expression of estrogen receptor (ER), progesterone receptor (PR), and human epidermal growth factor receptor 2 (HER2); and the Ki-67 index. Histological grading was assessed using the modified Bloom–Richardson system. Tumors were considered ER- and PR-positive if at least 1% of the tumor cells exhibited nuclear staining. Additionally, the database recorded various treatment factors such as anti-hormone therapy, HER2-targeted therapy, adjuvant chemotherapy, radiotherapy, and breast surgery. It also tracked oncologic outcomes, including survival rates, recurrence patterns, and mortality.

The project was reviewed and approved by the Institutional Review Board (IRB) of Yonsei University Severance Hospital (IRB no. 4-2024-1160). The need for informed consent was waived due to the retrospective nature of the study.

### 2.2. Treatment Protocols and Outcomes

All patients in this study underwent preoperative axillary ultrasound (AUS). Findings were classified as suspicious if they included any of the following: increased cortical thickness > 3 mm, complete loss of the lymph node (LN) hilum fat, tumor invasion throughout the LN hilum, extracapsular extension of LN involvement, or the presence of microcalcifications within the LN. A low axillary burden was defined as 1–2 metastatic LNs, while a high axillary burden indicated >2 metastatic LNs, as determined by the final pathological report. If ALN metastasis was detected through SLNB, ALND was selectively omitted based on the surgeon’s discretion and the patient’s condition, adhering to the Z0011 criteria. The study data were analyzed retrospectively to assess overall survival (OS) and recurrence-free survival (RFS) among the participants. OS was measured from the date of surgery until the occurrence of death from any cause. RFS was defined as the time from surgery to the first documented recurrence, which could occur locally, regionally, or at a distant site. Second primary malignancies, contralateral breast cancer, and deaths without signs of active disease were considered censoring events. Local recurrence was defined as the return of cancer at or near the primary tumor site, such as the skin, pectoral muscle, chest wall, surgical area, or another quadrant of the same breast. Regional recurrence was identified when cancer reappeared in the ipsilateral axillary, supraclavicular, or internal mammary lymph nodes.

The primary outcome of this study was to establish the differences in clinical manifestations between ILC and IDC. As a secondary outcome, the study compared survival rates based on histological differences.

### 2.3. Data and Statistical Analysis

Continuous clinicopathological variables were transformed into binary or multinomial categories based on medical evidence or their distribution to facilitate analysis. For these categorical variables, chi-square or Fisher’s exact tests were used to assess group differences. Univariate and multivariate logistic regression analyses were conducted to identify factors associated with ALN metastasis. Survival curves were estimated using the Kaplan–Meier method, and differences between groups were evaluated with the log-rank test. Hazard ratios (HRs) and 95% confidence intervals (CIs) were calculated for each variable using a Cox univariate model. To identify factors influencing survival outcomes between groups, multivariate Cox proportional hazards regression models were employed, accounting for interactions among significant variables. All statistical analyses were conducted using the SPSS software (version 29.0, IBM Corp., Armonk, NY, USA).

## 3. Results

### 3.1. Patient Characteristics

**Figure 1:**
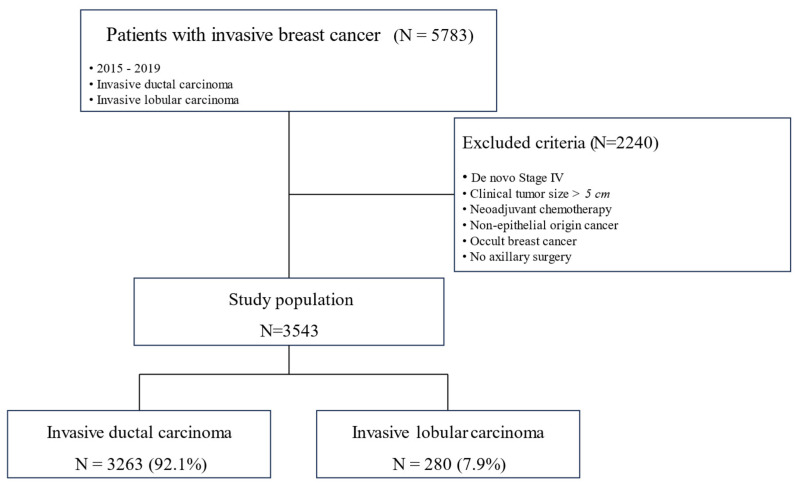
Scope of study population.

This study included 3543 subjects, 3263 (92.1%) of whom were diagnosed with IDC and 280 (7.9%) with ILC (Figure 1). Table 1 compares the clinicopathological characteristics of the two groups.

In this cohort of patients with early-stage breast cancer who underwent AUS and upfront surgery, ILC was more frequently observed in younger patients, showing a statistically significant difference (*p* = 0.004). The proportion of patients with multifocal or multicentric lesions was marginally higher in the ILC group; however, this difference was not statistically significant (*p* = 0.146). In contrast, a significant difference was observed in tumor size, with ILC being diagnosed at larger sizes (*p* < 0.001).

Traditional clinicopathological factors associated with poor prognosis were more frequently observed in the IDC group. Lymphovascular invasion (LVI) was more commonly detected in patients with IDC (*p* = 0.039). The proportion of HG 3 was also higher in IDC (22.7%) compared to ILC (5.0%) (*p* < 0.001). The Ki-67 index was also higher in IDC (*p* < 0.001). Finally, while most ILC cases were classified as the Luminal A subtype, IDC showed a greater proportion of more aggressive subtypes, including HER2 overexpression and triple-negative, compared to ILC (*p* < 0.001). The characteristics, treatment, and outcomes according to breast cancer subtypes and histology were described in Supplementary Table S1. Among ILC patients, 5 out of 44 (11.4%) were classified as Luminal B-like (HER2-positive), while among IDC patients, 314 (32.8%) fell into this category. Most Luminal B-like patients with ILC exhibited unfavorable factors such as old age, larger tumor sizes, high Ki-67, high grade, multifocality, and LVI.

### 3.2. Axillary Lymph Node Metastasis

**Figure 2:**
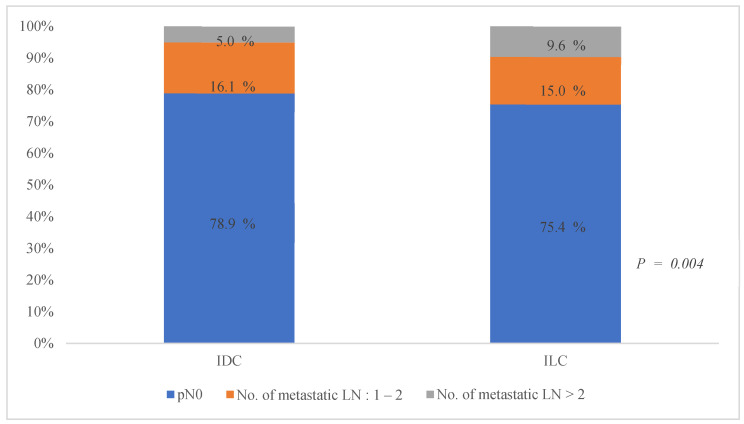
Proportions of metastatic lymph nodes according to histological types in early breast cancer. IDC, invasive ductal carcinoma; ILC, invasive lobular carcinoma; No., number; LN, lymph node.

The AUS showed no significant difference between the two groups in the proportion of suspicious findings (ILC: 14.9%, IDC: 13.9%, *p* = 0.655) or confirmed LN metastasis on final pathology (ILC: 24.6%, IDC: 21.1%, *p* = 0.172). However, an analysis of LN metastasis patterns revealed that a significantly higher proportion of patients with ILC were classified as having N2 or N3 stages, indicating a greater axillary burden (*p* < 0.001). Low axillary burden was similar between the groups, but a notable difference was observed in high axillary burden (Figure 2).

In the univariate analysis, factors significantly associated with LN metastasis included multifocality/multicentricity (*p* = 0.001), suspicious findings on preoperative AUS (*p* < 0.001), T2 (*p* < 0.001), LVI (*p* < 0.001), and higher HG (*p* = 0.021) (Table 2). In the multivariate analysis, factors that remained significant for LN metastasis were suspicious findings on AUS (*p* < 0.001), T2 (*p* < 0.001), and LVI (*p* < 0.001) (Table 3). For cases with > 2 LN metastases, the univariate analysis identified ILC (*p* = 0.001), suspicious findings on AUS (*p* < 0.001), T2 (*p* < 0.001), and LVI (*p* < 0.001) as significant factors. Multivariate analysis confirmed the same factors—ILC (*p* = 0.003), suspicious findings on AUS (*p* < 0.001), T2 (*p* < 0.001), and LVI (*p* < 0.001)—as significant. In the subgroup analysis based on AUS findings, no significant difference in axillary nodal burden was observed between IDC and ILC in the no suspicious findings group. However, a statistically significant difference was found in the suspicious findings group (Supplementary Figure S1).

The histological type was not significantly associated with LN metastasis (*p* = 0.172). However, when metastasis occurred, patients with ILC had a higher axillary burden (*p* < 0.001), indicating that although the incidence of metastasis is similar between ILC and IDC, ILC patients tend to have more ALN involvement.

### 3.3. Treatment and Survival Outcome According to Breast Cancer Histology

Compared to IDC, ILC presented with larger tumor sizes and a higher nodal burden, resulting in significant differences in the final TNM stage between the two groups (*p* < 0.001). Although the rate of ALN metastasis did not differ between the groups, patients with ILC were more likely to undergo ALND. Despite the higher stage, ILC showed fewer traditional clinicopathological factors associated with poor prognosis. Additionally, a higher proportion of ILC cases were classified as Luminal A subtype, leading to a lower rate of adjuvant chemotherapy compared with IDC (Supplementary Table S1).

**Figure 3:**
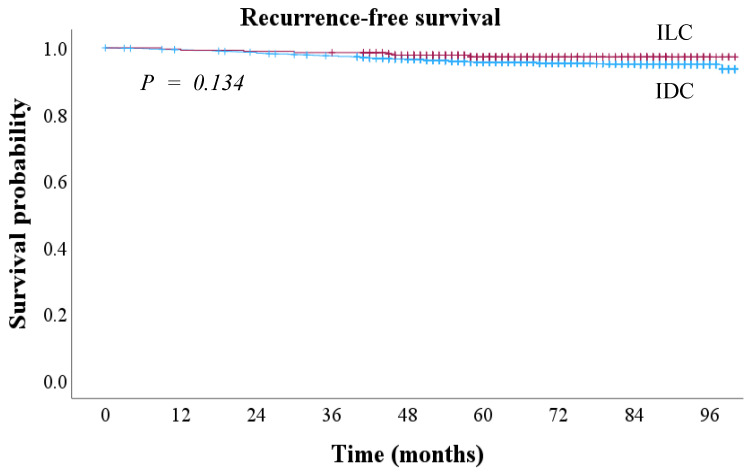
Recurrence-free survival outcomes according to histological types in early breast cancer. IDC, invasive ductal carcinoma; ILC, invasive lobular carcinoma.

**Figure 4:**
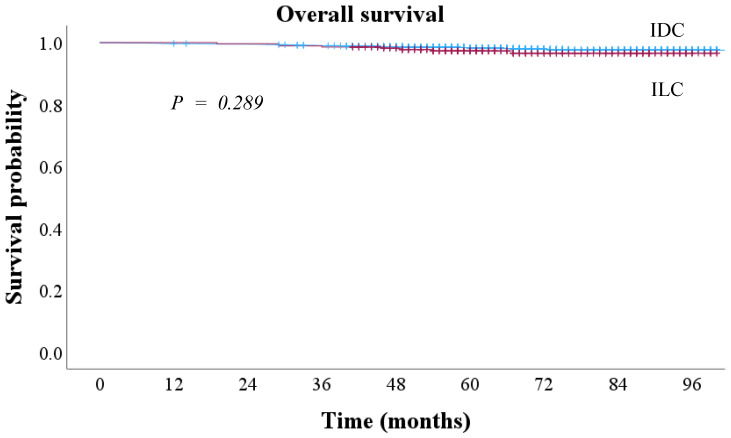
Overall survival outcomes according to histological types in early breast cancer. IDC, invasive ductal carcinoma; ILC, invasive lobular carcinoma.

The median follow-up period was 65 months. The 8-year RFS was 94.1% in IDC and 95.2% in ILC, with no statistically significant difference between the two groups (*p* = 0.134) (Figure 3). The 5-year OS was 97.4% in IDC and 97.1% in ILC, showing no significant difference (*p* = 0.289) (Figure 4).

RFS was analyzed using both univariate and multivariate analyses. The univariate analysis identified several factors significantly associated with RFS, including T stage, N stage, LVI, HG, Ki-67 index, and subtype. In the multivariate analysis, independent predictors of worse RFS included N stage (HR = 1.624, 95% CI: 1.125–2.345, *p* = 0.011) and HG 2 and 3 (HR = 2.481, 95% CI: 1.304–4.716, *p* = 0.006) (Table 4).

OS was analyzed using univariate and multivariate analyses. In the univariate analysis, significant factors associated with worse OS included age, T stage, N stage, HG, Ki-67, and subtype. Multivariate analysis identified age > 50 years (HR = 1.832, 95% CI: 1.101–3.049, *p* = 0.021), T2 stage (HR = 1.681, 95% CI: 1.002–2.821, *p* = 0.049), LN metastasis (HR = 1.953, 95% CI: 1.161–3.285, *p* = 0.012), and HG 2 and 3 (HR = 2.997, 95% CI: 1.168–7.693, *p* = 0.022) as independent predictors of mortality (Table 5). Histological type was not associated with survival in early-stage breast cancer.

## 4. Discussion

De-escalation strategies are increasingly being adopted in the treatment of early-stage breast cancer [13]. As the knowledge of breast cancer has grown, SLNB has replaced ALND as the standard axillary procedure in early breast cancer cases [14]. Additionally, ALND can be omitted in selected patients with ALN metastasis, and studies have shown that oncological safety is maintained even when SLNB is omitted in some early breast cancer cases [3,15]. In this study, we aimed to identify patient groups eligible for axillary surgery de-escalation in ILC by comparing its clinical characteristics and long-term outcomes with those of IDC.

This study compared the clinical characteristics and long-term survival of ILC and IDC, finding that, despite distinct features at diagnosis, RFS and OS outcomes were similar in both cases. ILC was typically diagnosed with larger tumor size and higher nodal burden, correlating with a more advanced TNM stage. However, with favorable clinicopathological factors and appropriate systemic therapy, no significant survival differences were observed, aligning with previous studies [16]. Importantly, there was no difference in the proportion of suspicious ALNs on preoperative AUS or the rate of nodal metastasis. However, when metastasis occurred, ILC exhibited more extensive spread in cases with ALN involvement.

According to previous studies, the LN metastasis patterns differ between ILC and IDC [17,18]. ILC is characterized by uniformly distributed cells within the LN when metastasis occurs, without distinct morphological changes [19,20]. Additionally, even in the presence of LN metastases, tissue destruction is not prominent, and the metastatic cells remain uniformly spread throughout the node. As a result, ILC metastatic cells tend to exhibit low nuclear atypia, small size, and a resemblance to surrounding lymphocytes [21,22,23,24]. The metastatic pattern of ILC observed in our study was consistent with previously reported findings. Furthermore, our study showed that histological differences did not affect LN metastasis in cases with negative AUS findings. Additionally, histology was not statistically significant in predicting LN metastasis or survival outcomes. These findings indicate that the likelihood of node metastasis does not increase in ILC compared with IDC. However, when LN metastasis occurs, ILC exhibits more extensive nodal involvement in the AUS suspicious findings group.

Landmark RCTs such as NSABP B-32, AMAROS, IBCSG 23-01, and Z0011 have established guidelines for axillary surgery in early breast cancer [3,25,26]. These RCTs demonstrated that, if SLNB was negative, ALND was unnecessary in selective patients. The Z0011 trial marked a pivotal turning point in axillary surgery for early breast cancer, and current clinical practice is based on the surgical approaches established by its findings. In the Z0011 trial, after adjusting for adjuvant therapy, age, and tumor type (ductal vs. lobular vs. other), the adjusted HR for OS between the SLNB-alone and ALND groups was 0.87 (90% CI: 0.62–1.23). A non-inferiority *p*-value of 0.03 confirmed that SLNB-alone treatment is not inferior to ALND in terms of survival. Although the study adjusted for variables such as adjuvant therapy, age, and tumor type, the number of patients with ILC was only 63 (7.36%); thus, caution is needed when interpreting the results for ILC, as the small sample size limits the statistical reliability of the subgroup analysis. Additionally, in this study, cN0 is defined as no palpable mass, and this should be carefully considered in clinical applications. The SINODAR-ONE trial included 130 ILC patients (14.79%) [27]. However, the relatively short median follow-up period (34 months) warrants caution.

According to our data, patients with ILC presented at more advanced stages and were younger compared to those with IDC. Among young, premenopausal women, systemic chemotherapy is typically administered conservatively. However, most ILC cases were classified as the Luminal A subtype and exhibited less aggressive prognostic factors. Consequently, this led to a lower adjuvant systemic chemotherapy rate than in IDC cases. Furthermore, histology did not significantly affect recurrence or mortality outcomes. Although LN metastasis remains a critical prognostic factor influencing both treatment decisions and survival, our findings indicate that when treatment strategies are appropriately tailored based on patient characteristics, cancer subtype, and other prognostic factors, there are no significant differences in survival outcomes between ILC and IDC cases. As most ILC cases in this study were classified under the Luminal A subtype, it is crucial to consult the findings of the MINDACT and RxPONDER trials when assessing the need for adjuvant chemotherapy [28,29]. Both trials included patients with up to three positive LNs, most of which underwent ALND. Previous studies have reported that, in patients with pathological node-positive disease, the presence and number of metastatic LNs did not significantly influence the outcomes of the Oncotype DX Recurrence Score or MammaPrint results [30]. For patients with ILC, omitting ALND based on Z0011 criteria may increase the risk of underestimating the axillary burden. In such cases, it is essential to carefully evaluate whether endocrine therapy alone can achieve favorable outcomes, as suggested by studies that included patients with up to three positive LNs.

CDK4/6 inhibitors should be considered in treating ILC, especially because many cases belong to the Luminal A subtype. The NATALEE and monarchE trials have demonstrated the efficacy of CDK4/6 inhibitors in early breast cancer [31,32]. The NATALEE trial included patients with stage II or higher cancer, considering factors like grade, Ki-67 index, and multigene assay results. Notably, 17.7% of participants were patients with ILC. Although interim analysis showed a 3.3% absolute benefit in reducing recurrence and death with ribociclib plus a non-steroidal aromatase inhibitor (NSAI) compared with NSAI alone, further follow-up is required to confirm these results. On the other hand, the monarchE trial included patients with ≥4 positive LNs or 1–3 positive nodes with tumors ≥ 5 cm or grade 3. However, ILC cases may miss eligibility if the de-escalation of axillary surgery reduces the detection of positive nodes.

Our study had some limitations. First, as a retrospective study, there may be selection bias. The study design restricted our ability to accurately assess the effectiveness of specific treatment methods. While ILC and IDC showed similar rates of ALN metastasis, patients with ILC were more likely to undergo further dissection. Although many patients met the Z0011 criteria, considering the metastatic patterns of ALNs in ILC, most node-positive cases still underwent ALND based on the surgeon’s preference. This makes it challenging to evaluate the impact of different extents of axillary surgery on ILC outcomes within the scope of our study. Second, due to the study design and IRB approval restrictions, it was not possible to determine the number of suspicious LNs. It is known that the more suspicious LNs identified in preoperative imaging, the higher the number of metastatic LNs detected postoperatively [33,34,35,36]. However, analyzing this aspect was challenging. Furthermore, the retrospective nature of the study made it difficult to accurately assess the effects of specific chemotherapy and endocrine therapy modalities. Nevertheless, our study is meaningful as it includes a broad range of patient groups across different ages, breast cancer subtypes, and anatomical stages of early breast cancer. The study focused on the long-term follow-up of patients with IDC and ILC who underwent upfront surgery, and the results demonstrate that adherence to treatment guidelines leads to excellent prognoses.

## 5. Conclusions

In conclusion, ILC in early breast cancer with AUS suspicious findings tends to be larger than IDC and, when metastasizing to LN, it involves a higher number of nodes. Therefore, caution is required when considering the reduction in axillary surgery in some ILC cases of early breast cancer. However, most cases are classified as Luminal A, and the clinicopathological factors indicating prognosis, such as grade and Ki-67 index, are generally more favorable in ILC compared with IDC. As a result, when appropriate treatment is administered, there is no difference in survival rates based on histological classification, and excellent survival outcomes are achieved.

## Figures and Tables

**Table 1:** Clinicopathological characteristics by histological type (invasive ductal carcinoma vs. invasive lobular carcinoma).

	Invasive Ductal Carcinoma	Invasive Lobular Carcinoma	*p*-Value
	(N = 3263)	(N = 280)	
Age			0.004
Age ≤ 50 years	1454 (44.6)	150 (53.6)	
Age > 50 years	1809 (55.4)	130 (46.4)	
Multicentricity			0.146
Unifocality	3109 (95.3)	261 (93.2)	
Multifocality/multicentricity	154 (4.7)	19 (6.8)	
Preoperative axillary ultrasound			0.655
No suspicious finding	2805 (85.9)	238 (85.1)	
Suspicious finding	458 (13.9)	42 (14.9)	
T stage			<0.001
T1	2675 (81.9)	192 (68.6)	
T2	588 (18.1)	88 (31.4)	
ALN metastasis			0.172
N0	2573 (78.9)	211 (75.4)	
Node metastasis	690 (21.1)	69 (24.6)	
N stage			<0.001
N0	2573 (78.9)	211 (75.4)	
N1mi	114 (3.5)	6 (2.1)	
N1	465 (14.3)	41 (14.6)	
N2	79 (2.4)	12 (4.3)	
N3	32 (0.9)	10 (3.6)	
TNM Stage			<0.001
I	2305 (70.6)	169 (69.8)	
II	847 (26.0)	89 (31.8)	
III	111 (3.4)	22 (7.9)	
Lymphovascular invasion			0.039
Negative	2956 (90.6)	264 (94.3)	
Positive	307 (9.4)	16 (5.7)	
Histological Grade			<0.001
Grade 1	778 (23.8)	28 (10.0)	
Grade 2	1744 (53.4)	238 (85.0)	
Grade 3	741 (22.7)	14 (5.0)	
Ki-67 index			<0.001
Ki-67 ≤ 20	1903 (58.5)	233 (83.5)	
Ki-67 > 20	1350 (41.5)	46 (16.5)	
Subtype			<0.001
Luminal A-like	1673 (51.3)	226 (80.7)	
Luminal B-like	956 (29.3)	44 (15.7)	
HER2 overexpression	295 (9.0)	3 (1.1)	
Triple-negative	339 (10.4)	7 (2.5)	
Breast surgery			<0.001
Breast-conserving surgery	2055 (63.1)	145 (51.8)	
Mastectomy	1208 (36.9)	135 (48.2)	
Axillar operation			0.021
SLNB	2724 (83.5)	218 (77.9)	
SLNB + ALND	539 (16.5)	62 (22.1)	
Retrieved sentinel lymph nodes	3.00 [1.00–4.00]	3.00 [1.00–5.00]	0.355
Positive sentinel lymph nodes	0.00 [0.00–1.00]	0.00 [0.00–1.00]	0.309
Retrieved total lymph nodes	4.00 [2.00–7.00]	4.00 [2.00–8.75]	0.016
Positive total lymph nodes	0.00 [0.00–1.00]	0.00 [0.00–2.00]	0.082
Adjuvant Chemotherapy	1558 (47.7)	105 (37.5)	<0.001
Radiation treatment	1938 (59.4)	161 (57.5)	0.793

HER2, human epidermal growth factor receptor 2; SLNB, sentinel lymph node biopsy; ALND, axillary lymph node dissection.

**Table 2:** Clinicopathological factors associated with axillary lymph node metastasis.

	Univariate Analysis		Multivariate Analysis	
	OR (95% CI)	*p*-Value	OR (95% CI)	*p*-Value
Histology				
Invasive ductal carcinoma	reference			
Invasive lobular carcinoma	1.219 (0.917–1.621)	0.172		
Multicentricity				
Unifocality	reference		reference	
Multifocality/multicentricity	1.715 (1.231–2.391)	0.001	1.353 (0.936–1.955)	0.108
Preoperative axillary ultrasound				
No suspicious finding	reference		reference	
Suspicious finding	3.884 (3.182–4.741)	<0.001	3.461 (2.776–4.315)	<0.001
T stage				
T1	reference		reference	
T2	3.586 (2.991–4.301)	<0.001	2.819 (2.305–3.446)	<0.001
Lymphovascular invasion				
Negative	reference		reference	
Positive	4.963 (3.921–6.284)	<0.001	3.958 (3.065–5.111)	<0.001
Histological Grade				
Grade 1	reference		reference	
Grades 2 and 3	1.265 (1.036–1.544)	0.021	0.843 (0.679–1.047)	0.122
Ki-67 index				
Ki-67 ≤ 20	reference			
Ki-67 > 20	1.082 (0.918–1.274)	0.348		
Subtype				
Luminal A-like	reference		reference	
Luminal B-like	1.297 (1.085–1.551)	0.004	0.921 (0.748–1.131)	0.425
HER2 overexpression	0.534 (0.374–0.764)	<0.001	0.385 (0.261–0.571)	<0.001
Triple-negative	0.632 (0.461–0.867)	0.004	0.473 (0.334–0.671)	<0.001

OR, odds ratio; CI, confidence interval; HER2, human epidermal growth factor receptor 2.

**Table 3:** Clinicopathological factors associated with more than two axillary lymph node metastases.

	Univariate Analysis		Multivariate Analysis	
	OR (95% CI)	*p*-Value	OR (95% CI)	*p*-Value
Histology				
Invasive ductal carcinoma	reference		reference	
Invasive lobular carcinoma	2.017 (1.316–3.091)	0.001	2.057 (1.278–3.312)	0.003
Multicentricity				
Unifocality	reference			
Multifocality/multicentricity	1.457 (0.812–2.615)	0.207		
Preoperative axillary ultrasound				
No suspicious finding	reference		reference	
Suspicious finding	7.919 (5.853–10.715)	<0.001	5.807 (4.188–8.053)	<0.001
T stage				
T1	reference		reference	
T2	5.677 (4.212–7.651)	<0.001	3.238 (2.328–4.502)	<0.001
Lymphovascular invasion				
Negative	reference		reference	
Positive	6.898 (5.001–9.516)	<0.001	5.329 (3.723–7.629)	<0.001
Histological Grade				
Grade 1	reference		reference	
Grades 2 and 3	1.925 (1.263–2.933)	0.002	1.026 (0.648–1.625)	0.913
Ki-67 index				
Ki-67 ≤ 20	reference			
Ki-67 > 20	1.312 (0.978–1.761)	0.07		
Subtype				
Luminal A-like	reference			
Luminal B-like	1.606 (1.169–2.207)	0.003		
HER2 overexpression	0.917 (0.506–1.662)	0.775		
Triple-negative	0.848 (0.477–1.506)	0.848		

OR, odds ratio; CI, confidence interval; HER2, human epidermal growth factor receptor 2.

**Table 4:** Univariate and multivariate analysis of relapse-free survival in the entire patient cohort.

Relapse-Free Survival				
	Univariate Analysis		Multivariate Analysis	
	HR (95% CI)	*p*-Value	HR (95% CI)	*p*-Value
Age				
Age ≤ 50 years	reference			
Age > 50 years	0.841 (0.611–1.156)	0.284		
Histology				
Invasive ductal carcinoma	reference			
Invasive lobular carcinoma	0.564 (0.264–1.205)	0.139		
Multicentricity				
Unifocality	reference			
Multifocality/multicentricity	0.857 (0.458–1.913)	0.857		
T stage				
T1	reference		reference	
T2	1.901 (1.343–2.691)	<0.001	1.318 (0.914–1.899)	0.139
N stage				
N0	reference		reference	
N+	1.809 (1.286–2.543)	<0.001	1.624 (1.125–2.345)	0.011
Lymphovascular invasion				
Negative	reference		reference	
Positive	1.716 (1.091–2.697)	0.019	1.277 (0.793–2.059)	0.315
Histological Grade				
Grade 1	reference		reference	
Grades 2 and 3	3.689 (1.996–6.815)	<0.001	2.481 (1.304–4.716)	0.006
Ki-67 index				
Ki-67 ≤ 20	reference		reference	
Ki-67 > 20	2.413 (1.739–3.348)	<0.001	1.531 (0.791–2.959)	0.205
Subtype				
Luminal A-like	reference		reference	
Luminal B-like	2.307 (1.581–3.369)	<0.001	1.202 (0.585–2.471)	0.616
HER2 overexpression	2.323 (1.287–3.871)	0.004	1.303 (0.586–2.898)	0.516
Triple-negative	2.725 (1.686–4.406)	<0.001	1.639 (0.771–3.484)	0.199

HR, hazard ratio; CI, confidence interval; HER2, human epidermal growth factor receptor 2.

**Table 5:** Univariate and multivariate analysis of overall survival in the entire patient cohort.

Overall Survival				
	Univariate Analysis		Multivariate Analysis	
	HR (95% CI)	*p*-Value	HR (95% CI)	*p*-Value
Age				
Age ≤ 50	reference		reference	
Age > 50	1.787 (1.079–2.959)	0.024	1.832 (1.101–3.049)	0.021
Histology				
Invasive ductal carcinoma	reference			
Invasive lobular carcinoma	1.486 (0.712–3.103)	0.292		
Multicentricity				
Unifocality	reference			
Multifocality/multicentricity	1.447 (0.624–3.357)	0.391		
T stage				
T1	reference		reference	
T2	2.357 (1.446–3.844)	<0.001	1.681 (1.002–2.821)	0.049
N stage				
N0	reference		reference	
Node positive	2.199 (1.354–3.571)	0.001	1.953 (1.161–3.285)	0.012
Lymphovascular invasion				
Negative	reference		reference	
Positive	1.281 (0.614–2.676)	0.509	0.874 (0.405–1.883)	0.731
Histological Grade				
Grade 1	reference		reference	
Grades 2 and 3	3.703 (1.491–9.197)	0.005	2.997 (1.168–7.693)	0.022
Ki-67 index				
Ki-67 ≤ 20	reference		reference	
Ki-67 > 20	1.669 (1.044–2.667)	0.032	1.173 (0.455–3.024)	0.741
Subtype				
Luminal A-like	reference		reference	
Luminal B-like	1.697 (0.994–2.997)	0.053	1.161 (0.421–3.196)	0.773
HER2 overexpression	0.659 (0.201–2.162)	0.491	0.492 (0.118–2.051)	0.331
Triple-negative	2.378 (1.236–4.575)	0.009	1.847 (0.656–5.195)	0.245

HR, hazard ratio; CI, confidence interval; HER2, human epidermal growth factor receptor 2.

## Data Availability

Some information can be provided upon request to the corresponding author. Data are contained within the article and Supplementary Materials.

## Supplementary Materials

The following supporting information can be downloaded at: https://www.mdpi.com/article/10.3390/cancers17061002/s1, Figure S1: The proportions of metastatic lymph nodes by histological type in a subgroup analysis based on preoperative axillary ultrasound; Table S1: Clinicopathological characteristics and treatment accordinsg to subtypes and histology.

## References

[1] Halsted W.S. (1894). The results of operations for the cure of cancer of the breast performed at the Johns Hopkins Hospital from June, 1889, to January, 1894. Ann. Surg..

[2] Fisher B., Montague E., Redmond C., Barton B., Borland D., Fisher E.R., Deutsch M., Schwarz G., Margolese R., Donegan W. (1977). Comparison of radical mastectomy with alternative treatments for primary breast cancer: A first report of results from a prospective randomized clinical trial. Cancer.

[3] Giuliano A.E., McCall L., Beitsch P., Whitworth P.W., Blumencranz P., Leitch A.M., Saha S., Hunt K.K., Morrow M., Ballman K. (2010). Locoregional recurrence after sentinel lymph node dissection with or without axillary dissection in patients with sentinel lymph node metastases: The american college of surgeons oncology group z0011 randomized trial. Ann. Surg..

[4] Li C.I., Anderson B.O., Daling J.R., Moe R.E. (2003). Trends in incidence rates of invasive lobular and ductal breast carcinoma. JAMA.

[5] Yoon K.H., Park S., Kim J.Y., Park H.S., Kim S.I., Cho Y.U., Park B.-W. (2019). Is the frozen section examination for sentinel lymph node necessary in early breast cancer patients?. Ann. Surg. Treat. Res..

[6] Gruel N., Lucchesi C., Raynal V., Rodrigues M.J., Pierron G., Goudefroye R., Cottu P., Reyal F., Sastre-Garau X., Fourquet A. (2010). Lobular invasive carcinoma of the breast is a molecular entity distinct from luminal invasive ductal carcinoma. Eur. J. Cancer.

[7] De Schepper M., Koorman T., Richard F., Christgen M., Vincent-Salomon A., Schnitt S.J., van Diest P.J., Zels G., Mertens F., Maetens M. (2024). Integration of pathological criteria and immunohistochemical evaluation for invasive lobular carcinoma diagnosis: Recommendations from the european lobular breast cancer consortium. Modern Path.

[8] Sastre-Garau X., Jouve M., Asselain B., Vincent-Salomon A., Beuzeboc P., Dorval T., Durand J.C., Fourquet A., Pouillart P. (1996). Infiltrating lobular carcinoma of the breast: Clinicopathologic analysis of 975 cases with reference to data on conservative therapy and metastatic patterns. Cancer.

[9] Ferlicot S., Vincent-Salomon A., Medioni J., Genin P., Rosty C., Sigal-Zafrani B., Freneaux P., Jouve M., Thiery J.-P., Sastre-Garau X. (2004). Wide metastatic spreading in infiltrating lobular carcinoma of the breast. Eur. J. Cancer.

[10] Van Baelen K., Geukens T., Maetens M., Tjan-Heijnen V., Lord C.J., Linn S., Bidard F.C., Richard F., Yang W.W., Steele R.E. (2022). Current and future diagnostic and treatment strategies for patients with invasive lobular breast cancer. Ann. Oncol..

[11] Weigelt B., Geyer F.C., Natrajan R., Lopez-Garcia M.A., Ahmad A.S., Savage K., Kreike B., Reis-Filho J.S. (2010). The molecular underpinning of lobular histological growth pattern: A genome-wide transcriptomic analysis of invasive lobular carcinomas and grade-and molecular subtype-matched invasive ductal carcinomas of no special type. J. Pathol..

[12] Sikora M.J., Cooper K.L., Bahreini A., Luthra S., Wang G., Chandran U.R., Davidson N.E., Dabbs D.J., Welm A.L., Oesterreich S. (2014). Invasive lobular carcinoma cell lines are characterized by unique estrogen-mediated gene expression patterns and altered tamoxifen response. Cancer Res..

[13] NCCN Clinical Practice Guidelines in Oncology (NCCN Guidelines®) Breast Cancer. Version 4.2024–10 August 2024. https://www.nccn.org/professionals/physician_gls/pdf/breast.pdf.

[14] Gentile D., Tinterri C. (2024). Sentinel lymph node biopsy versus axillary lymph node dissection in breast cancer patients undergoing mastectomy. Minerva Surg..

[15] Gentilini O.D., Botteri E., Sangalli C., Galimberti V., Porpiglia M., Agresti R., Luini A., Viale G., Cassano E., Peradze N. (2023). Sentinel lymph node biopsy vs no axillary surgery in patients with small breast cancer and negative results on ultrasonography of axillary lymph nodes: The sound randomized clinical trial. JAMA Oncol..

[16] Cipolla C., Lupo S., Grassi N., Tutino G., Greco M., Eleonora D.A., Gebbia V., Valerio M.R. (2024). Correlation between sentinel lymph node biopsy and non-sentinel lymph node metastasis in patients with cn0 breast carcinoma: Comparison of invasive ductal carcinoma and invasive lobular carcinoma. World J. Surg. Oncol..

[17] Carleton N., Oesterreich S., Marroquin O.C., Diego E.J., Tseng G.C., Lee A.V., McAuliffe P.F. (2022). Is the choosing wisely recommendation for omission of sentinel lymph node biopsy applicable for invasive lobular carcinoma?. Ann. Surg. Oncol..

[18] Grossi S., Le J., Armani A. (2023). Omitting axillary staging in selected patients: Rationale of choosing wisely in breast cancer treatment. Surgery.

[19] Lee J., Ku G.Y., Lee H., Park H.S., Ku J.S., Kim J.Y., Park S., Park B.-W. (2022). Lobular carcinoma in situ during preoperative biopsy and the rate of upgrade. Cancer Res. Treat..

[20] Narbe U., Bendahl P.-O., Fernö M., Ingvar C., Dihge L., Rydén L. (2021). St gallen 2019 guidelines understage the axilla in lobular breast cancer: A population-based study. Br. J. Surg..

[21] Topps A., Clay V., Absar M., Howe M., Lim Y., Johnson R., Bundred N. (2014). The sensitivity of pre-operative axillary staging in breast cancer: Comparison of invasive lobular and ductal carcinoma. Eur. J. Surg. Oncol..

[22] Biglia N., Maggiorotto F., Liberale V., Bounous V., Sgro L., Pecchio S., D’Alonzo M., Ponzone R. (2013). Clinical-pathologic features, long term-outcome and surgical treatment in a large series of patients with invasive lobular carcinoma (ilc) and invasive ductal carcinoma (idc). Eur. J. Surg. Oncol..

[23] Corona S., Bortul M., Scomersi S., Bigal C., Bottin C., Zanconati F., Fox S., Giudici F., Generali D. (2020). Management of the axilla in breast cancer: Outcome analysis in a series of ductal versus lobular invasive cancers. Breast Cancer Res. Treat..

[24] Boughey J.C., Middleton L.P., Harker L., Garrett B., Fornage B., Hunt K.K., Babiera G.V., Dempsey P., Bedrosian I. (2007). Utility of ultrasound and fine-needle aspiration biopsy of the axilla in the assessment of invasive lobular carcinoma of the breast. Am. J. Surg..

[25] Krag D.N., Anderson S.J., Julian T.B., Brown A.M., Harlow S.P., Costantino J.P., Ashikaga T., Weaver D.L., Mamounas E.P., Jalovec L.M. (2010). Sentinel-lymph-node resection compared with conventional axillary-lymph-node dissection in clinically node-negative patients with breast cancer: Overall survival findings from the nsabp b-32 randomised phase 3 trial. Lancet Oncol..

[26] Donker M., van Tienhoven G., Straver M.E., Meijnen P., van de Velde C.J., Mansel R.E., Cataliotti L., Westenberg A.H., Klinkenbijl J.H., Orzalesi L. (2014). Radiotherapy or surgery of the axilla after a positive sentinel node in breast cancer (eortc 10981-22023 amaros): A randomised, multicentre, open-label, phase 3 non-inferiority trial. Lancet Oncol..

[27] Tinterri C., Gentile D., Gatzemeier W., Sagona A., Barbieri E., Testori A., Errico V., Bottini A., Marrazzo E., Dani C. (2022). Preservation of axillary lymph nodes compared with complete dissection in t1–2 breast cancer patients presenting one or two metastatic sentinel lymph nodes: The sinodar-one multicenter randomized clinical trial. Ann. Surg. Oncol..

[28] Piccart M., van’t Veer L.J., Poncet C., Cardozo J.M.L., Delaloge S., Pierga J.-Y., Vuylsteke P., Brain E., Vrijaldenhoven S., Neijenhuis P.A. (2021). 70-gene signature as an aid for treatment decisions in early breast cancer: Updated results of the phase 3 randomised mindact trial with an exploratory analysis by age. Lancet Oncol..

[29] Kalinsky K., Barlow W.E., Gralow J.R., Meric-Bernstam F., Albain K.S., Hayes D.F., Lin N.U., Perez E.A., Goldstein L.J., Chia S.K. (2021). 21-gene assay to inform chemotherapy benefit in node-positive breast cancer. N. Engl. J. Med..

[30] Yoon K.H., Lee S.J., Kim Y., Ahn J.H., Kim J.Y., Park H.S., Kim S.I., Park S. (2023). A simplified risk scoring system for predicting high-risk groups in gene expression tests for patients with estrogen receptor-positive, human epidermal growth factor receptor 2-negative, and node-positive breast cancer. Ann. Surg. Treat. Res..

[31] Johnston S.R., Harbeck N., Hegg R., Toi M., Martin M., Shao Z.M., Zhang Q.Y., Martinez Rodriguez J.L., Campone M., Hamilton E. (2020). Abemaciclib combined with endocrine therapy for the adjuvant treatment of hr+, her2−, node-positive, high-risk, early breast cancer (monarche). J. Clin. Oncol..

[32] Slamon D., Lipatov O., Nowecki Z., McAndrew N., Kukielka-Budny B., Stroyakovskiy D., Yardley D.A., Huang C.-S., Fasching P.A., Crown J. (2024). Ribociclib plus endocrine therapy in early breast cancer. N. Engl. J. Med..

[33] Yoo T.-K., Kang B.J., Kim S.H., Song B.J., Ahn J., Park W.-C., Chae B.J. (2020). Axillary lymph node dissection is not obligatory in breast cancer patients with biopsy-proven axillary lymph node metastasis. Breast Cancer Res. Treat..

[34] Zhao X., Yang L., Cao C., Song Z. (2024). The prognostic analysis of further axillary dissection in breast cancer with 1-2 positive sentinel lymph nodes undergoing mastectomy. Front. Oncol..

[35] Heidinger M., Knauer M., Tausch C., Weber W.P. (2023). Tailored axillary surgery–a novel concept for clinically node positive breast cancer. Breast.

[36] Sinner H.F., Naffouje S., Selfridge J.M., Lee M.C., Hoover S.J., Laronga C. (2022). Surgical management of the axilla in invasive lobular carcinoma in the z1071 era: A propensity-score matched analysis of the national cancer database. Curr. Oncol..

