# Supplementary material for: Impact of Axillary Burden on Survival: A Comparative Study of Invasive Lobular Carcinoma and Invasive Ductal Carcinoma in Early-Stage Breast Cancer
Source: Cancers (Basel). 2025 Mar 17;17(6):1002. doi: 10.3390/cancers17061002 (PMC11940394; doi:10.3390/cancers17061002)
Supplement: Supplementary file 1 [file cancers-17-01002-s001.zip › cancers-3479359-supplementary.pdf]

Supplementary Table S1. Clinicopathological characteristics and treatment according to subtypes and histology

Subtype	Luminal A-like		Luminal B-like		HER2 overexpression		Triple-negative	
Histology	IDC (N = 1673)	ILC (N = 226)	IDC (N = 956)	ILC (N = 44)	IDC (N = 295)	ILC (N = 3)	IDC (N = 339)	ILC (N = 7)
Age								
Age ≤ 50	719 (43.0)	128 (56.6)	507 (53.0)	20 (45.5)	108 (36.6)	0	120 (35.4)	2 (28.6)
Age > 50	954 (57.0)	98 (43.4)	449 (47.0)	24 (54.5)	187 (63.4)	3 (100)	219 (64.6)	5 (71.4)
Multicentricity								
Unifocality	1593 (95.2)	212 (93.8)	920 (96.2)	39 (88.6)	277 (93.9)	3 (100)	319 (94.1)	7 (100)
Multifocality/multicentricity	80 (4.8)	14 (6.2)	36 (3.8)	5 (11.4)	18 (6.1)	0	20 (5.9)	0
T stage								
T1	1469 (87.8)	170 (75.2)	700 (73.2)	17 (38.6)	241 (81.7)	1 (33.3)	285 (78.2)	4 (57.1)
T2	204 (12.2)	56 (24.8)	256 (26.8)	27 (61.4)	53 (18.3)	2 (66.7)	74 (21.8)	3 (42.9)
N stage								
N0	1320 (78.9)	171 (75.5)	706 (73.8)	32 (72.7)	258 (87.5)	2 (66.7)	289 (85.3)	6 (85.7)
N1mi	63 (3.8)	5 (2.2)	36 (3.8)	1 (2.3)	7 (2.4)	0	8 (2.4)	0
N1	240 (14.3)	34 (15.0)	169 (17.7)	6 (13.6)	23 (7.8)	1 (33.3)	33 (9.7)	0
N2	40 (2.4)	11 (4.9)	29 (3.0)	1 (2.3)	5 (1.7)	0	5 (1.5)	0
N3	10 (0.6)	5 (2.2)	16 (1.7)	4 (9.1)	2 (0.7)	0	4 (1.2)	1 (14.3)
Lymphovascular invasion								
Negative	1541 (92.1)	213 (94.2)	818 (85.6)	41 (93.2)	278 (94.2)	3 (100)	319 (94.1)	7 (100)
Positive	132 (7.9)	13 (5.8)	138 (14.4)	3 (6.8)	17 (5.8)	0	20 (5.9)	0
Histologic Grade								
Grade1	675 (40.3)	27 (11.9)	88 (9.2)	1 (2.3)	2 (0.7)	0	13 (3.8)	0
Grade 2	956 (57.1)	198 (87.6)	541 (56.5)	35 (79.5)	151 (51.2)	1 (33.3)	96 (28.3)	4 (57.1)
Grade 3	42 (2.5)	1 (0.4)	327 (34.2)	8 (18.2)	142 (48.1)	2 (66.7)	230 (67.8)	3 (42.9)
Ki67								
Ki67 <= 20	1673 (100)	226 (100)	114 (11.9)	2 (4.5)	55 (18.7)	0	61 (18.5)	5 (83.3)
Ki67 > 20	0	0	842 (88.1)	42 (95.5)	239 (81.3)	3 (100)	269 (81.5)	1 (16.7)
Breast operation								
Breast conserving surgery	1145 (68.4)	133 (54.0)	562 (58.8)	18 (40.9)	119 (40.3)	3 (100)	229 (67.6)	2 (28.6)
Mastectomy	528 (31.6)	104 (46.0)	394 (41.2)	26 (59.1)	176 (59.7)	0	110 (32.4)	5 (71.4)
Axillary operation								

SLNB	1414 (84.5)	178 (78.8)	765 (80.0)	31 (70.5)	250 (84.7)	3 (100)	295 (87.0)	6 (85.7)
ALND	259 (15.5)	48 (21.2)	191 (20.0)	13 (29.5)	45 (15.3)	0	44 (13.0)	1 (14.4)
Radiation treatment	1072 (64.1)	138 (61.1)	540 (56.5)	19 (43.2)	115 (39.0)	1 (33.3)	211 (62.2)	3 (42.9)
Endocrine treatment	1619 (96.8)	219 (96.9)	849 (96.7)	43 (97.7)				
HER2 targeted treatment			82.2 (258/314) *	60 (3/5) *	68.1 (201/295) *	100 (3/3) *		
Adjuvant Chemotherapy	414 (24.7)	67 (29.6)	660 (69.0)	31 (70.5)	213 (72.2)	3 (100)	271 (79.9)	4 (57.1)
Recurrence	45 (2.7)	5 (2.2)	56 (5.9)	2 (4.5)	17 (5.8)	0	25 (7.4)	0
Death	25 (1.5)	4 (1.8)	22 (2.3)	3 (6.8)	3 (1.0)	0	12 (3.5)	1 (14.3)

Unless otherwise indicated, data are the number of patients with percentages in parentheses for categorical variables.

* The data is presented as percentages, where within the parentheses, the denominator represents the number of patients indicated for each treatment, and the numerator denotes the number of patients who received the treatment.

SLNB, sentinel lymph node dissection; ALND, Axillary lymph node dissection; HER2, human epidermal growth factor receptor 2

Supplementary Figure S1. The proportions of metastatic lymph nodes by histological type in a subgroup analysis based on preoperative axillary ultrasound.

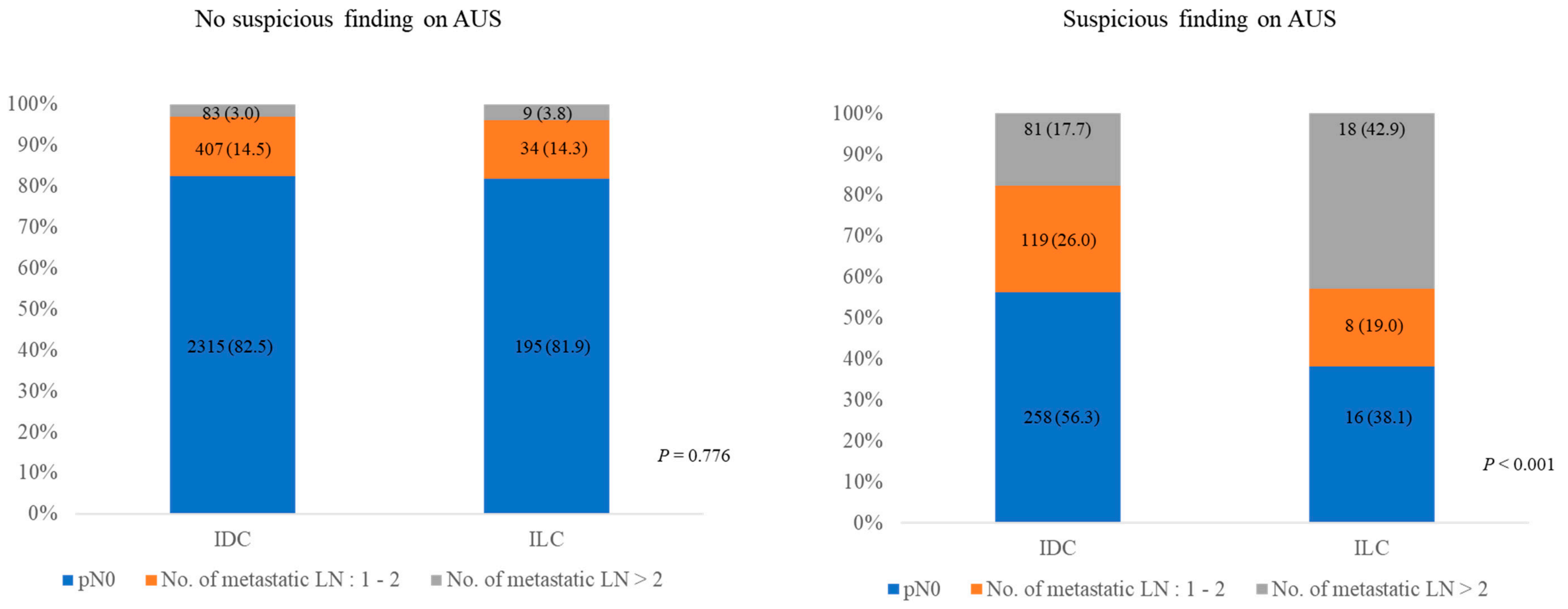

No., Number; LN, Lymph
